# Langerhan's Cell Histiocytosis of Sphenoid Sinus causing Vision Loss: A Case Report

**DOI:** 10.31729/jnma.3959

**Published:** 2019-08-31

**Authors:** Suresh Mani, Regi Thomas, John Mathew, Rajiv Michael

**Affiliations:** 1 Department of ENT, Christian Medical College, Vellore, India

**Keywords:** *blindness*, *histiocytosis*, *langerhans-cell*, *sphenoid sinus*

## Abstract

Langerhan's cell histiocytosis is an uncontrolled proliferation of dendritic cells. The involvement of skull base is rare. Variable clinical presentation and multi organ involvement often warrant a multidisciplinary approach for a successful diagnosis. We are reporting a case of 16-year-old male with sphenoid sinus Langerhan's cell histiocytosis which presented as a sudden and painless loss of vision. It is a rare entity in the diagnosis of blindness. Delayed diagnosis and treatment can result in serious complications. The radiological features and management options are discussed with a review of the pertinent literature.

## INTRODUCTION

In general, monocular vision loss usually indicates an ocular problem. Non ocular lesions also cause monocular vision loss when it arises from close structures. Non ocular lesions are easily missed due to its non specific symptoms as well as complex anatomical location. Sphenoid sinus lesions can cause vision loss due to its close relationship with optic nerve. Optic neuropathy from sphenoid sinus may arise from the spread of sinus inflammation and infection, compression by an expansible lesion, neoplasm or ischemia.^1–3^

The clinical presentation of sphenoid sinus lesions involves vague headaches and may be associated with purulent rhinorrhoea, retropharyngeal drip, nasal obstruction, abnormal vision and nerve deficit. Inflammatory conditions appear to be the major cause of sphenoid sinus lesions accounting for 65-72% of cases, followed by neoplasm (benign and malignant) accounting for 16 to 17.5%.^4,5^

Langerhan's cell histiocytosis (LCH) is a rare neoplasm and occurrence in the sphenoid sinus is very rare. Isolated sphenoid sinus LCH lesions most commonly present with headache, followed by ophthalmological and nasal symptoms. Delayed diagnosis may occur due to its nonspecific symptoms. Ophthalmologist plays a vital role in suspecting non ocular causes of vision loss when they find normal ophthalmic examination. High index of suspicion is required as endoscopic examination can be normal despite sphenoid sinus lesion.^4–6^ As our case had a rare tumor in an uncommon location with a vague presentation and normal nasal endoscopy, the diagnosis was made in a dilemma.

## CASE REPORT

A 16-year-old male presented with complaints of headache which was more on the right side and decreased vision in the right eye for one month. There was no history of trauma, sinusitis or any eye complaints in the past. Ear, nose and throat examination was unremarkable. Documented vision on right eye was 2/60 and relative afferent pupillary defect (RAPD) with normal left eye. Fundus/posterior segment examination were normal on both the sides. Extraocular movements were restricted on medial aspect on right eye grossly and not able to perform the visual field charting due to vision loss. Other Neurological examinations were normal and no signs of meningitis.

**Figure 1.**
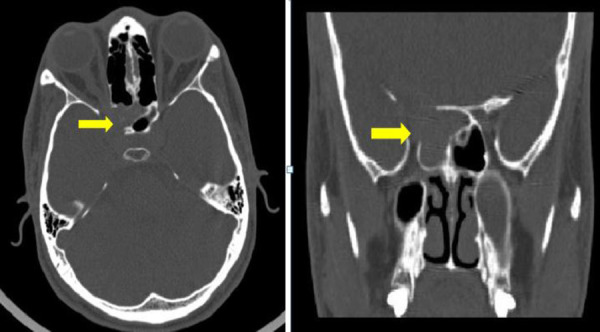
CT axial and coronal images showing the soft tissue with hyper dense foci seen in right sphenoid sinus with erosion of lateral wall and involving orbital apex.

CT showed ill-defined soft tissue with hyper dense foci seen in right sphenoid sinus with erosion of lateral wall and involving orbital apex (Figure 1).

**Figure 2.**
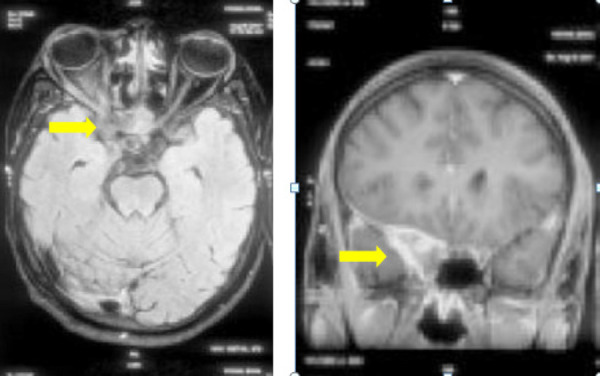
MRI T1W axial and Gad T1W coronal images showing the lesion enhancement in post gadolinium study.

In MRI, lesion appears hypointense in T2/FLAIR and intense enhancement in post gadolinium contrast study (Figure 2).

**Figure 3.**
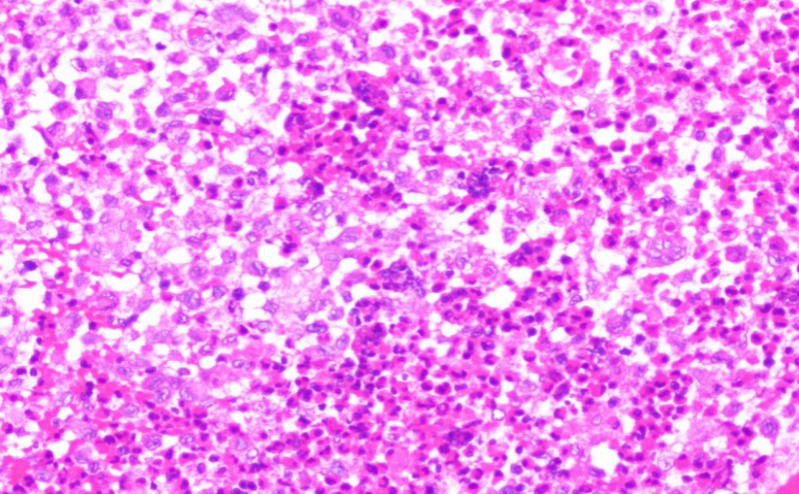
Hematoxylin and eosin 200× showing large foamy, multinucleated histiocytes with deep cleft with giant cells and mixed inflammatory cells.

**Figure 4.**
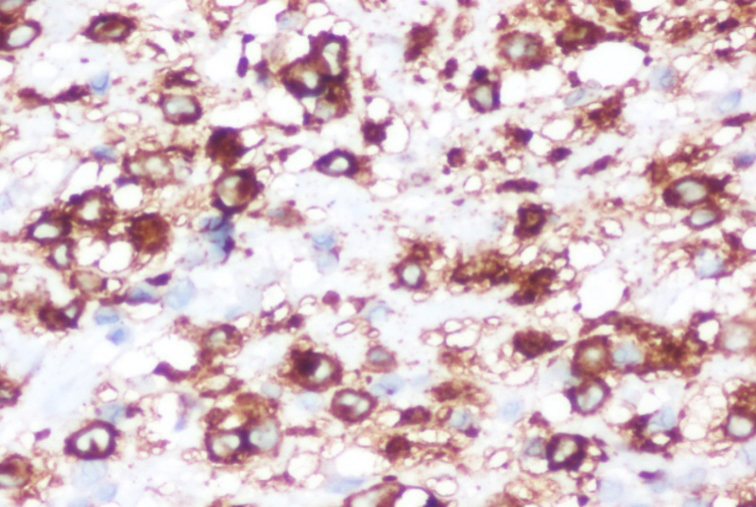
The tumor cells positive for CD1a immunostain (×400) suggestive of Langerhans cell histiocytosis.

We had differential diagnosis of invasive fungal sinusitis, lymphoma and malignant neoplasm. We were in a dilemma whether to treat him with an empirical anti-fungal therapy before histological and microbiological confirmation in view of rapid vision deterioration. We performed an emergency endoscopic debridement of the lesion under general anaesthesia and sent specimens for fungal KOH, culture and histopathological examination. KOH smear was negative for fungus and histological features were suggestive of Langerhan's cell histiocytosis. Diagnosis of LCH was confirmed by H&E stain showing highly cellular, polygonal histiocytes with round nuclei and CD1a antigenpositivity (Figure 3 and 4).

We referred him to a haematologist for further management. They performed a bone marrow biopsy which showed normal cellular marrow with no abnormal cells. Thus, the diagnosis of a solitary type LCH (SSLCH) was made. Unfortunately, he lost to follow up without taking treatment at our centre.

## DISCUSSION

LCH is an uncontrolled proliferation of dendritic cells with an undetermined aetiology. The clinical presentation of LCH is heterogeneous, ranging from mild disease with self-healing, solitary lesions (SS-LCH) to multisystem disease with fatal dissemination (MS-LCH) (liver, lung, spleen, or bone marrow). Solitary lesion is the most common and 75% of cases occur before 20 years of age. The involvement of skull base is very rare and the temporal bone is the most common site. The delayed diagnosis may be rarity of lesion in skull base and vague symptoms. Variable clinical presentation and multi organ involvement often warrant a multidisciplinary approach for successful diagnosis.^7,8^

In our patient, the solitary lesion extended to the sphenoid sinus, orbital apex, parasellar, sellar and the right cavernous sinus. In this region, the other radiological differential diagnosis that must be considered includes chordoma, chondroma, chondrosarcoma, meningioma, fibrous dysplasia, tubercular or fungalosteomyelitis, metastatic disease, primary Ewing's sarcoma, plasmacytoma, hemangioblastoma, giant cell tumor of the sphenoid.^6^

Vision loss is one of the serious complications of sphenoid sinus lesions. Most patients visit ophthalmologist for their vision loss, the examinations could be normal without any oblivious fundus changes and might look like primary optic neuritis.^3^ This may be the one of the reason for late diagnosis of retro orbital lesions. Whenever ophthalmologist encounters a vision loss patient with normal or mild fundus changes which is not correlating with vision loss, he/she should be sent to ENT / Neurosurgeon for evaluation of possible retro orbital causes.

Imaging in the form of CT / MRI with contrast should be done despite normal endoscopic nasal examination. Sphenoid sinus soft tissue in CT /MRI with vision loss should be biopsied to rule out possible fungal or neoplastic aetiology.^9^

Our patient had a MRI scan with contrast before visiting our hospital; this helped us to go ahead with an early endoscopic surgery. Intra operative frozen section and KOH smear study helped us in differentiating the fungal lesion from a neoplastic lesion as well as to avoid treating them with empirical anti-fungal therapy. In view of vision loss, most of the surgery was done in an emergency basis to prevent further deterioration of vision. The timing of surgery and the availability of these facility will improve the diagnostic approach as well as avoid empirical therapies.

Histopathological examination shows large foamy, multinucleated histiocytes with deep cleft with reactive infiltration. Diagnosis is confirmed if stains are positive for CD1a antigen or Birbeck granules seen with electron microscopy.^10^ Surgical excision is the treatment for most of SS LCH but this may be not applicable in head and neck region due to functional and cosmetic aspects. In skull base, complete surgical excision may not be possible in sphenoid sinus lesions due to involvement of cavernous part of internal carotid artery, cavernous sinus and optic nerve. In our case, debridement of the lesion was done as much as possible and mainly on the optic nerve part for possible compressive effect. Other treatment modalities like steroid therapy, chemotherapy and radiation therapy can be offered for residual lesions. The prognosis is better in SS LDH compared with MS LDH and has been improved because of newer chemotherapeutic agents which are much tolerable with fewer side effects.^10,11^

The limitations of this report are lack of outcome of the disease in this patient and follow up. Being a tertiary care centre, most of the patients travel from various places and not able to stay for long time due their financial issues as well as poor family support.

## Conflict of Interest:

**None.**

## Consent:

**JNMA Case Report Consent Form**was signed by the patient and the original is attached with the patient's chart.
